# Paradoxical embolism from a migrated right atrial ‘lead ghost’ through an unrecognized patent foramen ovale after cardiac resynchronization therapy–defibrillator lead extraction: a case report

**DOI:** 10.1093/ehjcr/ytag344

**Published:** 2026-05-08

**Authors:** Jaber Almohammad, Ahmed Almarzuqi, Jaimie Manlucu, Linrui Guo, Habib Rehman Khan

**Affiliations:** Cardiology Department, London Health Sciences Centre Research Institute, 339 Windermere Rd, London, ON N6A 5A5, Canada; Cardiology Department, London Health Sciences Centre Research Institute, 339 Windermere Rd, London, ON N6A 5A5, Canada; Cardiology Department, London Health Sciences Centre Research Institute, 339 Windermere Rd, London, ON N6A 5A5, Canada; Cardiology Department, London Health Sciences Centre Research Institute, 339 Windermere Rd, London, ON N6A 5A5, Canada; Cardiology Department, London Health Sciences Centre Research Institute, 339 Windermere Rd, London, ON N6A 5A5, Canada

**Keywords:** Lead ghost, Transvenous lead extraction, Patent foramen ovale, Paradoxical embolism, Cardiac resynchronization therapy-defibrillator, Stroke, Case report

## Abstract

**Background:**

A ‘lead ghost’ is a fibrotic or thrombotic cast that remains after transvenous lead extraction (TLE) and typically resides within the right heart. This case describes a paradoxical embolism through an unrecognized patent foramen ovale (PFO), most likely due to a transient right-to-left shunt that allowed the ‘lead ghost’ to enter the systemic circulation.

**Case summary:**

A 55-year-old man with ischaemic cardiomyopathy (ICM) underwent cardiac resynchronization therapy–defibrillator lead extraction for right-ventricular (RV) lead fracture. Intraoperative transoesophageal echocardiography revealed a 3.5-cm fibrous cast detaching from the right atrium (RA) and traversing an unrecognized PFO into the left atrium (LA). Surgical removal of the migrated cast with concomitant PFO closure was performed approximately 48 h after extraction, following multidisciplinary evaluation, as it remained unchanged in size despite therapeutic anticoagulation and a device-pocket hematoma. Approximately 24 h after surgery, he developed expressive aphasia; brain computed tomography (CT) confirmed a left middle cerebral artery (MCA) territory infarct without large-vessel occlusion. He was discharged to neuro-rehabilitation with mild residual aphasia.

**Discussion:**

This case highlights paradoxical embolism from a migrated RA ‘lead ghost’ through a PFO, a rare but high-impact complication of TLE. Transient right-to-left shunting may occur when RA pressure exceeds LA pressure during procedural conditions (e.g. positive-pressure ventilation or Valsalva). In this case, the PFO was not appreciated on initial intra-procedural imaging; subsequent echocardiography, including agitated-saline study with Valsalva, demonstrated right-to-left shunting. Definitive management was achieved with surgical extraction of the migrated cast and concomitant PFO closure.

Learning points

- Lead ghosts are common after lead extraction and usually remain in the right heart, but transient right-to-left shunting through a PFO can cause paradoxical embolism.
- Stroke from a migrated lead ghost is primarily structural rather than thrombotic and not prevented by antithrombotic therapy.
- Surgical removal with concomitant PFO closure provides definitive treatment and prevents recurrence.

## Introduction

Residual fibrotic sheaths or thrombi, termed ‘lead ghosts,’ have been reported in 8%–14% of patients in earlier series and up to 44% in large transoesophageal echocardiography (TOE)-guided cohorts.^1,2^ These structures are usually confined to the right heart and generally cause no symptoms, but in the presence of a patent foramen ovale (PFO), they can occasionally cross into the systemic circulation.^1,2^ Approximately 25% of the population has a PFO, which increases the risk of systemic embolization in patients with cardiac implantable electronic devices (CIEDs).^3,4^

We present a case of paradoxical embolism due to migration of a right atrium (RA) ‘lead ghost’ through a previously unrecognized PFO during cardiac resynchronization therapy–defibrillator (CRT-D) lead extraction. Although the PFO was not appreciated on early intra-procedural TOE, subsequent transthoracic echocardiography (TTE) with agitated saline contrast and Valsalva demonstrated provokable right-to-left shunting, supporting a paradoxical mechanism for embolization of the migrated cast.

## Summary figure

**Table:** 

Date/Time	Event
Jan 2, 2024	CRT-D alarm; device interrogation raised concern for RV lead failure → referred for TLE.
2007	TOE with agitated-saline study for suspected stroke/TIA → no right-to-left shunt detected.
Jan 12, 2024	Admitted for lead extraction planning (procedure scheduled for Jan 15).
Jan 15, 2024 (16:00)	Laser-assisted RV lead extraction was performed.
Jan 15, 2024 (post-procedure)	TOE demonstrated a linear echo-dense structure within the LA with a base at the interatrial septum, consistent with the migration of a fibrous cast with suspected thrombotic component through a previously unrecognized PFO → admitted to Coronary Care Unit (CCU) for neurological monitoring. IV UFH was initiated immediately; multidisciplinary discussion (EP, CS, IR) regarding definitive management.
Jan 16, 2024 (10:00)	TTE with agitated saline and Valsalva demonstrated right-to-left shunting, supporting functional PFO physiology. Experts’ input obtained regarding percutaneous aspiration/retrieval (including AngioVac) versus surgical extraction.
Jan 16–17, 2024	LA mass remained unchanged in size despite therapeutic anticoagulation, and a clinically significant device-pocket hematoma developed. After shared decision-making, operative management was favoured to enable definitive PFO closure and mitigate the risk of fragmentation/systemic embolization with percutaneous manipulation.
Jan 17, 2024 (10:00)	Minimally invasive surgical removal of the LA cast/thrombus with concomitant PFO closure, removal of RA fibrous tissue/thrombus, and exploration/management of the device-pocket hematoma. Cast/thrombus extracted from the PFO; three additional fibrous cast fragments identified in the RA and removed. Intra-operative TOE (colour Doppler) confirmed no residual interatrial shunt and no remaining cast/thrombus.
Jan 17, 2024 (post-operative period)	Therapeutic anticoagulation discontinued; transitioned to pharmacologic VTE prophylaxis (dalteparin 5,000 IU daily).
Jan 18, 2024 (11:00)	Expressive aphasia recognized; CT head confirmed a left MCA territory infarct involving the lateral left frontal lobe without LVO → not a candidate for endovascular treatment (EVT). Single antiplatelet therapy initiated per Neurology recommendations.
Jan 23, 2024	Post-closure follow-up TTE: interatrial septum intact with no residual interatrial shunt and no intracardiac thrombus.
Jan 26, 2024	Right-sided CRT-D re-implantation performed.
Jan 30, 2024	Discharged to stroke rehabilitation with persistent expressive aphasia and otherwise good functional recovery.

## Case presentation

**Figure 1:**
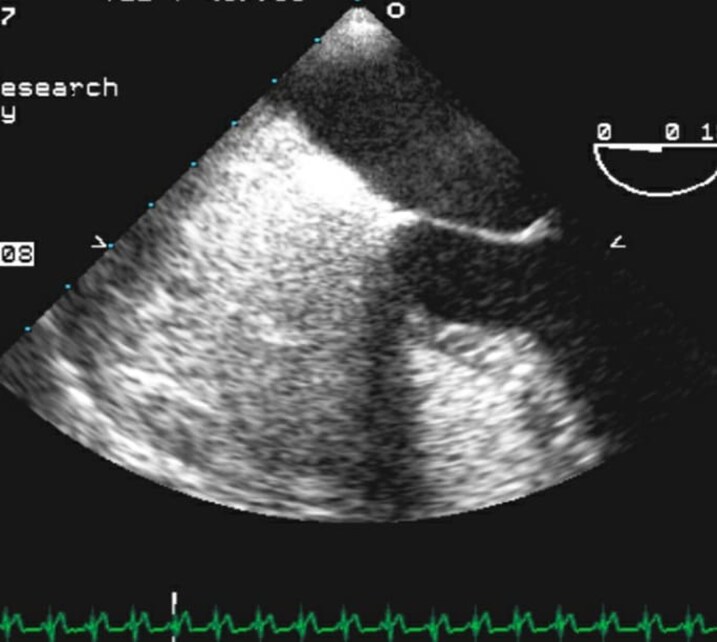
TOE from 2007 with agitated-saline (bubble) study performed during evaluation of suspected stroke/TIA, demonstrating no right-to-left interatrial shunt (no bubbles visualized in the left atrium).

**Figure 2:**
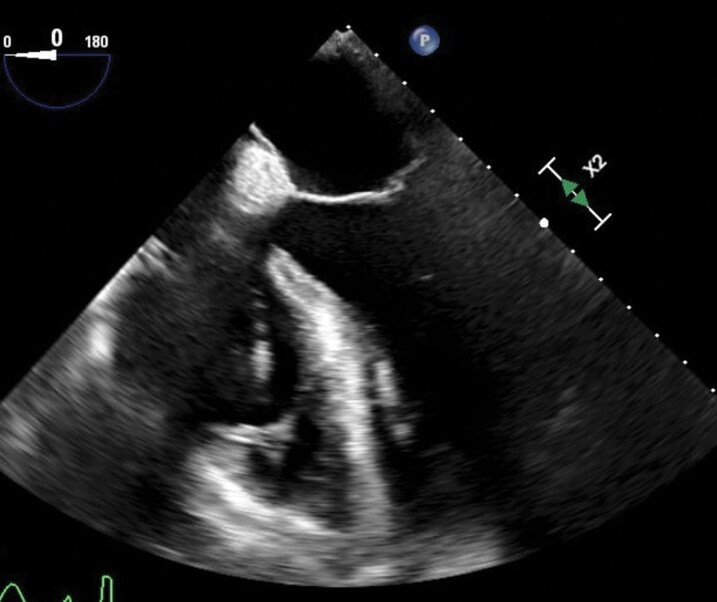
Baseline intraoperative TOE (2D) demonstrating the right-ventricular lead within the right heart, with no clearly visible lead-associated cast and no definitive PFO anatomy on standard imaging at that time.

A 55-year-old man with ischaemic cardiomyopathy (left ventricular ejection fraction 30%–35%), hypertension, dyslipidaemia, and prior CRT-D implantation presented with suspected RV lead fracture. He was not receiving chronic anticoagulation, and no pre-procedural anticoagulation was administered as there was no independent indication. A prior TOE with agitated-saline (bubble) study performed in 2007 during evaluation of suspected stroke/TIA showed no right-to-left interatrial shunt (no PFO detected) (*Figure 1*). Pre-extraction TTE and the initial intraoperative TOE assessment likewise did not identify a PFO or any lead-associated cast (*Figure 2*).

**Figure 3:**
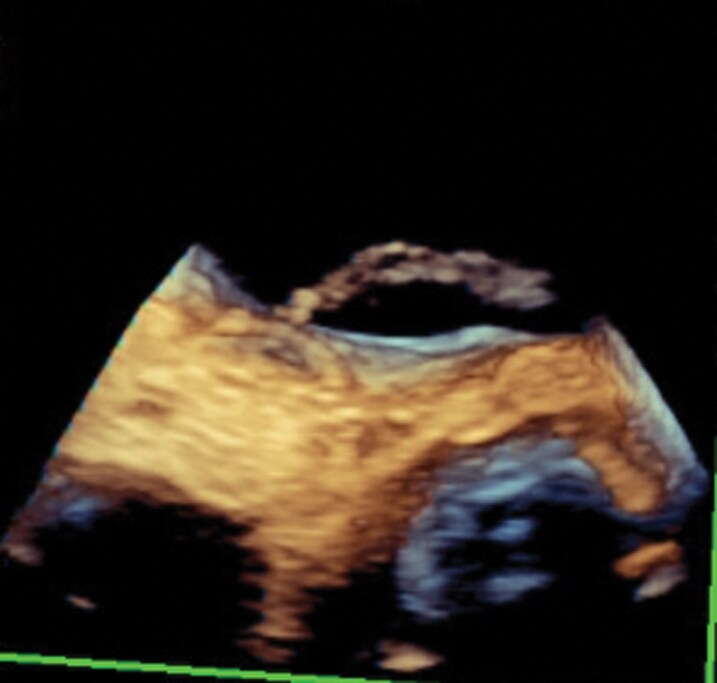
Intraoperative 3D TOE showing a lead-associated fibrous cast traversing the PFO into the left atrium.

However, during continuous intraoperative TOE monitoring throughout laser-assisted extraction under general anaesthesia, a 3.5–4 cm mobile echogenic structure in the right atrium consistent with a fibrous ‘lead ghost’ became apparent. During manipulation, the structure detached and was seen traversing a previously unrecognized PFO into the left atrium (*Figure 3*). On 16 January, post-extraction TTE with an agitated-saline bubble study and Valsalva demonstrated right-to-left shunting, confirming functional PFO physiology.

Given concern for thrombus layered on the migrated fibrous cast, intravenous (IV) unfractionated heparin (UFH) was initiated immediately after left-sided migration was identified (5000 units IV bolus followed by continuous infusion per institutional protocol) and titrated to a therapeutic aPTT target (60–79 s) to reduce thromboembolic risk. A multidisciplinary team (Electrophysiology, Cardiac Surgery, and Interventional Radiology) reviewed management options, including percutaneous aspiration/retrieval vs. operative extraction with concomitant PFO closure. Experts’ input was obtained on aspiration-based approaches (including AngioVac). Over approximately 48 h, the LA mass remained unchanged in size despite anticoagulation, and the patient developed a clinically significant device-pocket haematoma.

**Figure 4:**
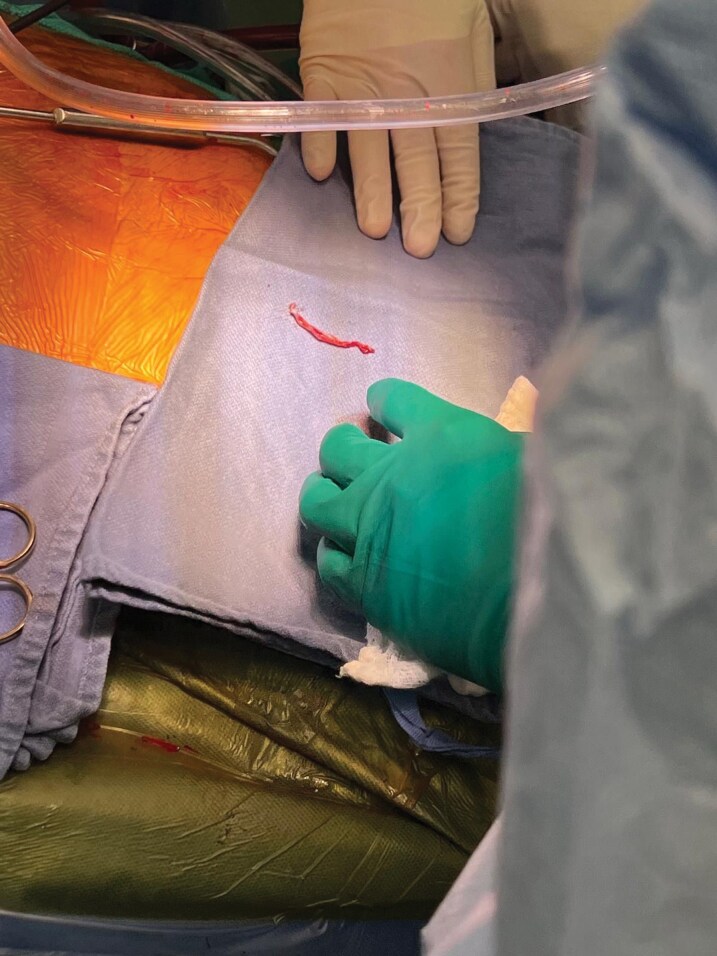
Gross photograph of the fibrous ‘lead ghost’ cast immediately after surgical removal.

**Figure 5:**
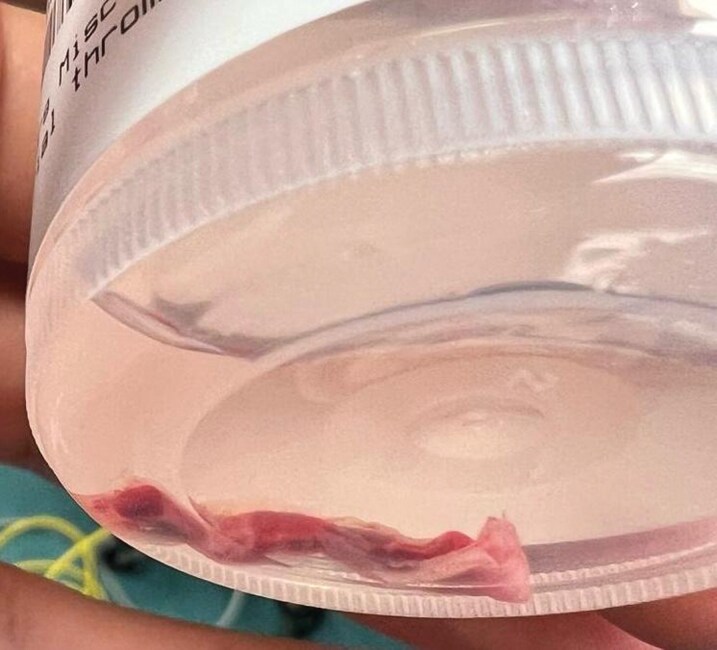
The extracted fibrous cast preserved in a specimen container.

After shared decision-making with the patient, operative management was favoured because of the large, mobile LA component, the need for definitive PFO closure, and concern that percutaneous manipulation could fragment the cast/thrombus and precipitate systemic embolization. Approximately 48 h after lead extraction, minimally invasive surgical removal of the LA cast/thrombus was performed with concomitant PFO closure, removal of RA fibrous tissue/thrombus, and management of the device-pocket haematoma. The cast/thrombus was extracted from the PFO and removed (*Figures 4* and *5*); three additional fibrous cast fragments were identified in the RA and removed as well. Intra-operative TOE with colour Doppler confirmed no residual interatrial shunt and that the cast/thrombus was no longer demonstrable.

Following surgery, therapeutic UFH was discontinued, and the patient was transitioned to pharmacologic venous thromboembolism (VTE) prophylaxis (dalteparin 5000 IU daily) and single antiplatelet therapy per Neurology recommendations. Approximately 24 h after surgery, the patient developed expressive aphasia; brain CT demonstrated a left MCA territory infarct involving the lateral left frontal lobe without large vessel occlusion (LVO), consistent with cardioembolic stroke. On Jan 23, follow-up TTE demonstrated an intact interatrial septum with no residual interatrial shunt and no intracardiac thrombus.

## Discussion

Lead ghosts are fibrotic encapsulations or organized thrombi that form around chronically implanted device leads.^1,5^ Two main types have been described: flying ghosts, which are mobile remnants that move within the RA or RV and typically disappear into the pulmonary circulation, and stable ghosts, which remain adherent to the endocardial surface or venous entry site after extraction.^2^ Most ghosts are harmless, but a flying ghost may rarely migrate into the systemic circulation if a PFO or other shunt is present.^2^

Under normal haemodynamic conditions, LA pressure exceeds RA pressure, resulting in left-to-right flow across a PFO when present; however, transient events can briefly reverse this gradient.^6^ Positive-pressure ventilation, endotracheal intubation, Valsalva manoeuvre, coughing, or straining can acutely raise RA pressure and open the foramen flap.^6,7^ Increased pulmonary vascular resistance during anaesthetic induction or mechanical ventilation may further promote transient right-to-left shunting.^7^ These short-lived reversals are well documented in PFO physiology.^6,7^

In this patient, embolic risk was primarily structural: a mobile intracardiac lead cast that migrated into the systemic circulation. Although anticoagulation may reduce thrombus propagation on the cast, it does not eliminate the hazard posed by a mobile foreign body within the left atrium. Once left-sided migration was identified, definitive source control (cast removal) and elimination of the conduit (PFO closure) were prioritized. Percutaneous aspiration/retrieval approaches (including AngioVac) were discussed with multiple specialists; however, surgical extraction was selected to minimize the risk of unpredictable fragmentation and systemic embolization, while enabling definitive PFO closure and addressing concomitant device-pocket complications. This case underscores the importance of pre-procedural PFO screening in selected high-risk patients and the value of continuous intraoperative TOE monitoring to promptly recognize migration or shunting events and guide management.

## Conclusion

Right-atrial lead ghosts are a frequent finding after TLE.^1,2^ However, systemic embolization is rare.^2^ When an unrecognized PFO exists, fibrotic debris can cross into the systemic circulation and cause disabling a stroke.^2,3^ Comprehensive echocardiographic evaluation, including colour Doppler interrogation and manoeuvres that transiently raise right-atrial pressure, may help identify clinically relevant shunting. When left-sided migration of a lead cast occurs, surgical removal with concomitant PFO closure can provide definitive treatment and reduce recurrence risk.^6,7^

## Patient perspective

The patient expressed relief that the cause of his stroke had been identified and definitively treated. He reported gradual improvement in speech function and remained highly motivated to continue rehabilitation, acknowledging the importance of the rapid multidisciplinary intervention in his recovery.

## Data Availability

The data underlying this article are available within the article and its accompanying figures. No additional datasets were generated or analysed.

## Lead author biography

Dr. Jaber Almohammad is a cardiology fellow at the London Health Sciences Centre and Research Institute, Western University, in London, Ontario, Canada. His clinical interests include cardiac electrophysiology, device management, and procedure-related complications. He is particularly interested in the diagnosis and management of device- and lead-related events. Dr. Almohammad is committed to developing expertise in electrophysiology and contributing to clinical practice through case-based learning, research engagement, and collaboration within the cardiovascular community.

The lead author affirms that this manuscript is an honest, accurate, and transparent account: no important aspects have been omitted.

## Notes

**Consent:** Written informed consent for publication of clinical details and images was obtained from the patient.
